# A role of human RNase P subunits, Rpp29 and Rpp21, in homology directed-repair of double-strand breaks

**DOI:** 10.1038/s41598-017-01185-6

**Published:** 2017-04-21

**Authors:** Enas R. Abu-Zhayia, Hanan Khoury-Haddad, Noga Guttmann-Raviv, Raphael Serruya, Nayef Jarrous, Nabieh Ayoub

**Affiliations:** 1 Department of Biology, Technion - Israel Institute of Technology, Haifa, 3200003 Israel; 2 Department of Microbiology and Molecular Genetics, IMRIC, The Hebrew University-Hadassah Medical School, 91120 Jerusalem, Israel

## Abstract

DNA damage response (DDR) is needed to repair damaged DNA for genomic integrity preservation. Defective DDR causes accumulation of deleterious mutations and DNA lesions that can lead to genomic instabilities and carcinogenesis. Identifying new players in the DDR, therefore, is essential to advance the understanding of the molecular mechanisms by which cells keep their genetic material intact. Here, we show that the core protein subunits Rpp29 and Rpp21 of human RNase P complex are implicated in DDR. We demonstrate that Rpp29 and Rpp21 depletion impairs double-strand break (DSB) repair by homology-directed repair (HDR), but has no deleterious effect on the integrity of non-homologous end joining. We also demonstrate that Rpp29 and Rpp21, but not Rpp14, Rpp25 and Rpp38, are rapidly and transiently recruited to laser-microirradiated sites. Rpp29 and Rpp21 bind poly ADP-ribose moieties and are recruited to DNA damage sites in a PARP1-dependent manner. Remarkably, depletion of the catalytic H1 RNA subunit diminishes their recruitment to laser-microirradiated regions. Moreover, RNase P activity is augmented after DNA damage in a PARP1-dependent manner. Altogether, our results describe a previously unrecognized function of the RNase P subunits, Rpp29 and Rpp21, in fine-tuning HDR of DSBs.

## Introduction

The human genome is susceptible to endogenous and exogenous DNA damaging agents^1, 2^. Failure to sense and repair DNA damages can lead to accumulation of mutations and genetic instability, thus increasing the chances of tumorigenesis^3, 4^. DNA damage induces rapid and highly orchestrated changes in chromatin structure that initiate the DNA damage response (DDR) and promote the accumulation of numerous DNA repair proteins at damaged sites^5–7^. Beside DDR proteins, emerging evidence implicates non-coding RNAs (ncRNAs) in DDR and tumorigenesis^8–12^. Various ncRNAs regulate the expression of DDR genes, such as ATM, BRCA1, H2AX and RAD51^13–16^. RNAs also serve as templates for DNA repair mechanisms^17, 18^. Moreover, DNA damage induces the expression of small and long ncRNAs, which regulate the recruitment of DDR proteins to chromatin and promote double-strand break (DSB) repair^19–21^.

DSBs are considered the most cytotoxic type of DNA damage, as a single unrepaired DSB can trigger cell death^22–25^. Vertebrate cells use at least two distinct pathways for DSB repair. The first is non-homologous end joining (NHEJ), an error-prone process that functions throughout the cell cycle. The second pathway is homology-directed repair (HDR), an error-free process that occurs in late S and G2 phases, in which a new chromatid is available and serves as a template for repair^26, 27^. Here, we unprecedentedly implicate the human RNase P protein subunits, Rpp29 and Rpp21, in HDR of DSBs.

Ribonuclease (RNase) P is an RNA enzyme that catalyzes the cleavage of the 5′ leader of precursor tRNA in the three domains of life, Bacteria, Archaea and Eukarya^28–30^. In human cells, nuclear RNase P has a catalytic RNA subunit, H1 RNA, associated with at least ten distinct protein subunits, termed Rpp14, Rpp20, Rpp21, Rpp25, Rpp29, Rpp30, Rpp38, Rpp40, hPop1 and hPop5^31–33^, some of which serve as cofactors in catalysis^34, 35^. Rpp21, Rpp29, Rpp30 and hPop5 are the core components of the catalytic ribonucleoprotein (RNP), as these proteins are conserved from Archaea to human^36^. Rpp20, Rpp21, Rpp25, Rpp29, Rpp30, Rpp38 and hPop5 directly bind to H1 RNA *in vitro* ^32, 37–39^. Biochemical studies further demonstrate that recombinant Rpp21 and Rpp29 are sufficient for reconstitution of the endonucleolytic activity of H1 RNA in processing of the 5′ leader of precursor tRNA *in vitro* ^34^. In addition to processing of precursor tRNA, human RNase P is involved in 3′-end maturation of long noncoding RNAs, such as MALAT1 and NEAT1^40–42^. H1 RNA is mainly found in the cell nucleoplasm, but is also localized in the nucleolus, perinucleolar compartment and cytoplasm^43, 44^. Many Rpp subunits are confined to nucleoli, which seem to serve as an assembly site, whereas other proteins are enriched in the nucleoplasm and Cajal bodies^31, 45–47^.

Human RNase P has non-canonical roles in regulating transcription of small ncRNA genes by RNA polymerase III (Pol III) and rRNA genes by RNA polymerase I (Pol I)^37, 48, 49^. Catalytic forms of this RNP exist in proficient initiation complexes assembled on target gene loci^50^. Human RNase P binds to chromatin of 5S rRNA and tRNA genes, as well as to rRNA gene repeats^37, 48–50^. In contrast to type I and type II genes transcribed exclusively by Pol III, the RPPH1 gene, a type III gene coding for the H1 RNA, shares a bi-directional promoter with the PARP2 gene and is transcribed by both Pol III and Pol II^51^, thus raising the concept that H1 RNA is linked to DDR. Of note, Matrin 3, a substrate of the ATM kinase, binds to RMRP RNA, the RNA subunit of the mitochondrial RNA processing ribonuclease, RNase MRP^52^, which has been derived from an ancestral RNase P RNA via RNA gene duplication and neofunctionalization^30^. In addition, proteomic screen reveals that ionizing radiation (IR)-induced DSBs trigger phosphorylation of Rpp29 on serine 10, possibly by the phosphatidylinositol 3-kinase-related kinases (PIKKs) family^53^. Collectively, the aforementioned data prompted us to assess whether human RNase P has a role in DDR.

## Results

### Rpp29 promotes homology-directed repair of double-strand breaks

**Figure 1:**
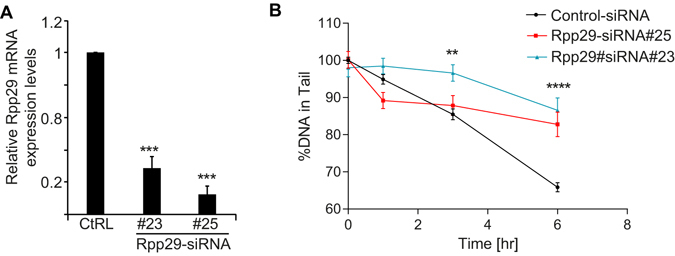
Rpp29 promotes repair of IR-induced DNA damage. (**A**) Real-time PCR analysis shows ~90% reduction in the steady state levels of Rpp29 mRNA in cells transfected with two different Rpp29 siRNAs, #23 and #25, and compared with those in cells transfected with control (CtRL) siRNA. P-values were calculated by two-sided Student’s t-test relative to ctrl siRNA; ***p < 0.001. (**B**) Control and Rpp29 depleted cells were exposed to 10 Gys of ionizing radiation (IR), harvested at the indicated time points after IR, and subjected to neutral comet assay. Quantitation of the DNA percentage in comet tail reveals DSB-repair deficiency in Rpp29 depleted cells. Error bars represent standard deviation (SD) from three independent experiments (n = 80 cells). Two-way ANOVA was used to test for differences at each dose; ** and **** indicate significance at p < 0.01 and p < 0.0001, respectively.

Mock and Rpp29-depleted U2OS cells were exposed to 10 Gys of IR and examined at different time points after damage by neutral comet assay (see Materials and Methods). Results demonstrate that Rpp29 knockdown using two different specific siRNAs (Fig. 1A) disrupted DSB repair, as evident from the high percentage of DNA in the comet tail, which reflected the levels of DSBs in individual cells. Indeed, the percentage of damaged DNA in the comet tail was significantly higher in Rpp29 deficient cells at 1, 3 and 6 h after IR when compared with that seen in mock-transfected cells (Fig. 1B). This outcome indicates that Rpp29 depletion increases the accumulation of broken DNA, possibly by reducing the IR-induced DSB repair.

**Figure 2:**
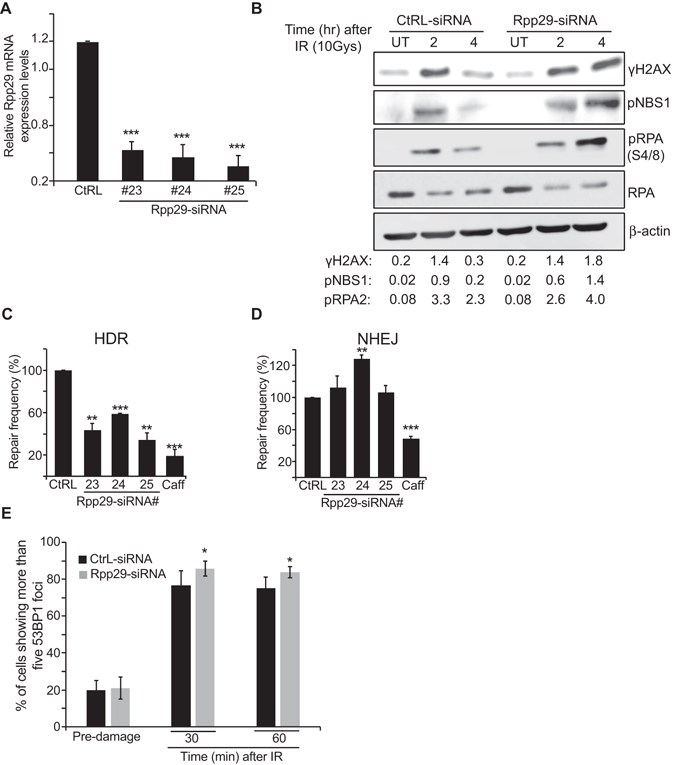
Rpp29 promotes homology-directed repair of double-strand breaks. (**A**) Quantitative real-time PCR shows the efficiency of Rpp29 knockdown in U2OS-TLR cells. Cells were transfected with control siRNA (Ctrl) or three different Rpp29 siRNAs. The y-axis represents the relative Rpp29 mRNA level normalized to that of GAPDH. P-values were calculated by two-sided Student’s t-test relative to Ctrl siRNA; ***p < 0.001. (**B**) Rpp29 knockdown increases IR-induced levels of pRPA2 S4/S8 phosphorylation, γH2AX, and NBS1 phosphorylation levels. Control and Rpp29-depleted U2OS cells were exposed to IR (10 Gys) and protein extracts were prepared at the indicated time points and subjected to western blot analysis using antibodies directed against the indicated proteins. Total RPA and β-actin were used as internal controls. The bands intensities of γH2AX, pNBS1 or pRPA were normalized relative to the intensities of their respective β-actin or total RPA bands, respectively. The ratios are shown at the bottom of the blot. Results are representative of two independent experiments. (**C**) Rpp29 knockdown impairs HDR of DSBs generated by I-SceI endonuclease. A reduction of ~50% in GFP-positive cells was observed after Rpp29 depletion. Caffeine was used as a positive control. Results shown are typical of three independent experiments. Error bars represent SD. P-values were calculated by two-sided Student’s t-test relative to Ctrl siRNA; ** and **** indicate significance at P < 0.01 and 0.0001. Given that HDR can occur only in S/G2 cell phase, data were corrected to flow-cytomertry S/G2 values. **(D)** TLR results for DSBs repair by NHEJ. Rpp29 knockdown had no significant effect on the integrity of NHEJ. Data represents SD for three independent experiments. **(E)** Percentage of control and Rpp29-depleted cells with more than five 53BP1 foci. Data shown represent at least 300 cells. Two-way ANOVA was used to test for differences at each dose; *p < 0.05.

To gain molecular insights into the mechanism by which Rpp29 affects DNA damage repair, we looked at the phosphorylation kinetics of early DNA damage markers. We found that the phosphorylation levels of two well-characterized ATM substrates, H2AX-Ser139 and NBS1-Ser343 were significantly increased in Rpp29-depleted cells compared to control cells (Fig. 2A,B). Similarly, RPA2 phosphorylation at Ser4 and Ser8 (pRPA) was greatly intensified in 4 h after damage induction, in Rpp29 depleted cells (Fig. 2B). Since phosphorylated RPA2 is a known indicator for ssDNA generation following DNA end resection, which promotes HDR of DSB^54, 55^, these site-specific phosphorylation support the existence of persistent DNA 5′-end due to defective HDR of DSBs.

To confirm a direct role of Rpp29 in DSB repair, we engineered a traffic light reporter (TLR) system^56^ integrated into the genome of U2OS cells (U2OS-TLR). This system enables simultaneous evaluation of DSB repair by HDR and NHEJ in the same cell (Fig. S1). Remarkably, Rpp29 depletion in U2OS-TLR cells using three different siRNAs resulted in ~50% decrease in the number of GFP-expressing cells, when compared with cells treated with control siRNA (Fig. 2C). This decrease demonstrates that Rpp29 is required for intact HDR of DSBs.

Interestingly, the above reduction of HDR was accompanied by an increase in mCherry production (Fig. 2D), an indication that Rpp29 depletion had no deleterious effect on the integrity of NHEJ. Apparently, the activation of NHEJ pathway is a counteracting response of the cells to elevated numbers of DSBs owing to the defective HDR. As a control, treatment of U2OS-TLR cells with caffeine, which is known to inhibit PIKKs^57^, brought to a severe reduction in DSB repair by both HDR and NHEJ (Fig. 2C,D). Furthermore, the enhanced NHEJ in cells deficient in Rpp29 was concomitant with a moderate increase in 53BP1 foci, thereby favoring DSB repair by NHEJ (Fig. 2E).

### Rpp29 and Rpp21 are rapidly and transiently recruited to laser-microirradiated DNA sites

**Figure 3:**
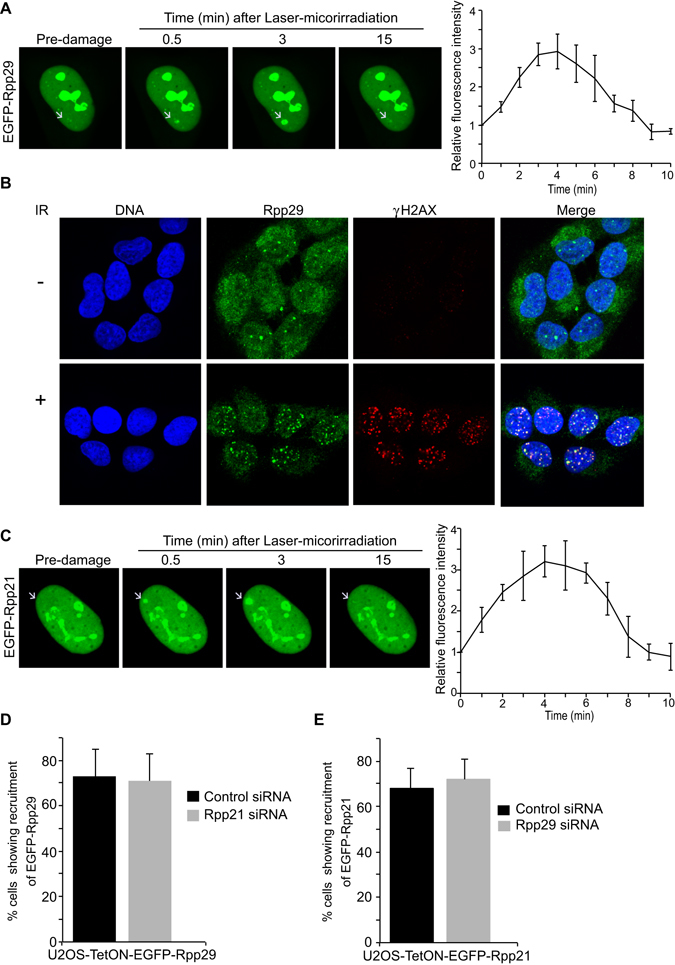
Rpp21 and Rpp29 show rapid and transient recruitment to DNA damage sites. (**A**) Time-lapse images show the localization of EGFP-Rpp29 at the indicated time points after laser micro-irradiation of a single region, marked by a white arrow. Graph (Right) shows fold increase in the relative fluorescence intensity of EGFP-Rpp29 at laser-microirradiated sites. Each measurement is representative of at least 10 cells. Error bars indicate SD. (**B**) Representative images showing IRIF enriched with endogenous Rpp29 protein. U2OS cells were exposed to IR (5 Gy), fixed after 3 min recovery and stained for DNA (blue), Rpp29 (green) and γH2AX (red). A merged image is seen on the right. Results shown are typical of three independent experiments and represent at least 15 different cells. (**C**) As in (**A**), time-lapse images showing the recruitment of EGFP-Rpp21 to laser microirradiated sites. (**D,E**) Graphs display the percentage of Rpp21 (**D**) and Rpp29 (**E**) deficient cells showing recruitment of EGFP-Rpp29 and EGFP-Rpp21 to damaged sites, respectively, as compared with cells transfected with control siRNA. Error bars represent the SEM from two independent experiments.

We sought to corroborate the role of Rpp29 in DDR by monitoring its subcellular localization before and after DNA damage induction. For this purpose, U2OS-TetON cell line that conditionally expresses EGFP-Rpp29 fusion protein upon the addition of doxycycline was established (Fig. S2). The cell line, termed U2OS-TetON-EGFP-Rpp29, was subjected to laser micro-irradiation that induces a localized DNA damage within the nucleus of living cells^58, 59^ and protein localization was monitored. Results show that ~70% of the laser-microirradiated cells (n = 50) exhibited rapid accumulation of EGFP-Rpp29 at laser-microirradiated sites. The accumulation was detectable within 15 seconds after irradiation, thus demonstrating that Rpp29 recruitment is an early event in the DDR. Accumulation of Rpp29 peaked at ~5 minutes and was followed by gradual dispersal out of the DNA damage sites (Fig. 3A).

To further substantiate the recruitment of Rpp29 to DNA damage sites, localization of the endogenous Rpp29 was monitored by immunofluorescence analysis after cells exposure to ionizing radiation. The results revealed that endogenous Rpp29 was mobilized to IR-induced foci (IRIF) and extensively co-localized with γH2AX foci (Fig. 3B). This co-localization at IRIF is consistent with localization of exogenous Rpp29 fused to GFP at laser-microirradiated sites, and confirms that this protein is targeted to DSBs.

Next, we sought to determine the effect of DNA damage on the subcellular localization of other subunits of RNase P, including its specific subunit Rpp21, which is not shared by RNase MRP^60^. Similar to Rpp29, Rpp21 also displayed rapid accumulation and dispersal kinetics at DNA damage sites (Fig. 3C). Of note, the recruitment of Rpp29 and Rpp21 to laser-microirradiated sites was independent of each other. Thus, knockdown of Rpp21 had no visible effect on Rpp29 accumulation at laser-microirradiated sites and vice versa (Fig. 3D,E). By contrast, Rpp14, Rpp25 and Rpp38, which are not components of the catalytic core of RNase P^38^, showed no perceptible recruitment to laser-microirradiated sites (Fig. S3). We concluded therefore that not all human RNase P protein subunits are recruited to DNA damage sites. The recruitment of Rpp21 to DNA damage sites prompted us to determine its effect on DSB repair using the above-described U2OS-TLR reported cells. Results show that depletion of Rpp21 using two different siRNAs (Fig. S4A) leads to ~50% decrease in GFP-positive cells compared to control siRNA-treated cells (Fig. S4B). On the other hand, Rpp21 depletion had no significant changes in the percentage of mCherry positive cells (Fig. S4C). Altogether, we concluded that, similar to Rpp29, Rpp21 predominantly facilitates HDR of DSB. Interestingly, Rpp21 and Rpp29 are amplified in many types of human cancer^61, 62^, we sought therefore to determine whether overexpression of Rpp21 and Rpp29 affects the integrity of DSB repair. Toward this end, U2OS-TLR cells were transfected with expression vectors encoding flag, flag-Rpp21 or flag-Rpp29 and the efficacy of DSB repair was determined (Fig. S5A). Results show that overexpression of neither Rpp21, nor Rpp29 has an effect on the integrity of HDR or NHEJ of DSBs (Fig. S5B,C).

### PARP1-depenedent recruitment of Rpp21/Rpp29 to DNA damage sites

**Figure 4:**
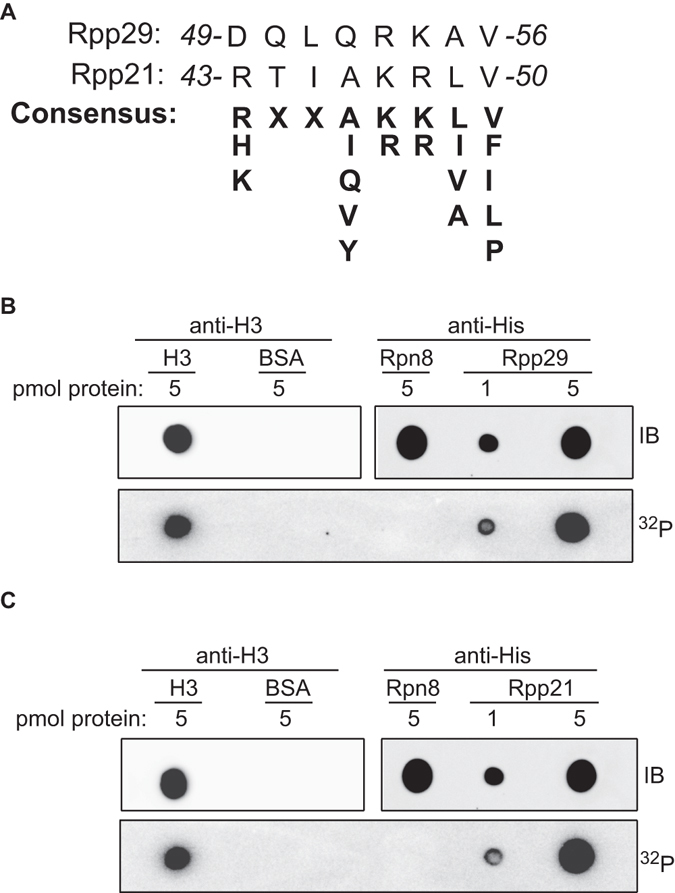
Rpp29 and Rpp21 bind poly(ADP-ribose) (PAR) *in vitro*. (**A**) Protein alignment reveals that Rpp29 and Rpp21 contain a PAR-binding consensus (bold letters). (**B**) PAR-binding assay with full-length, 6 × His-tagged recombinant Rpp29 and Rpp21 proteins. 6 × His-Rpn8 and BSA are shown as negative controls, whereas histone H3 is used as a positive control. IB: Immunoblot. ^32^P: phosphate radiolabeled PAR.

**Figure 5:**
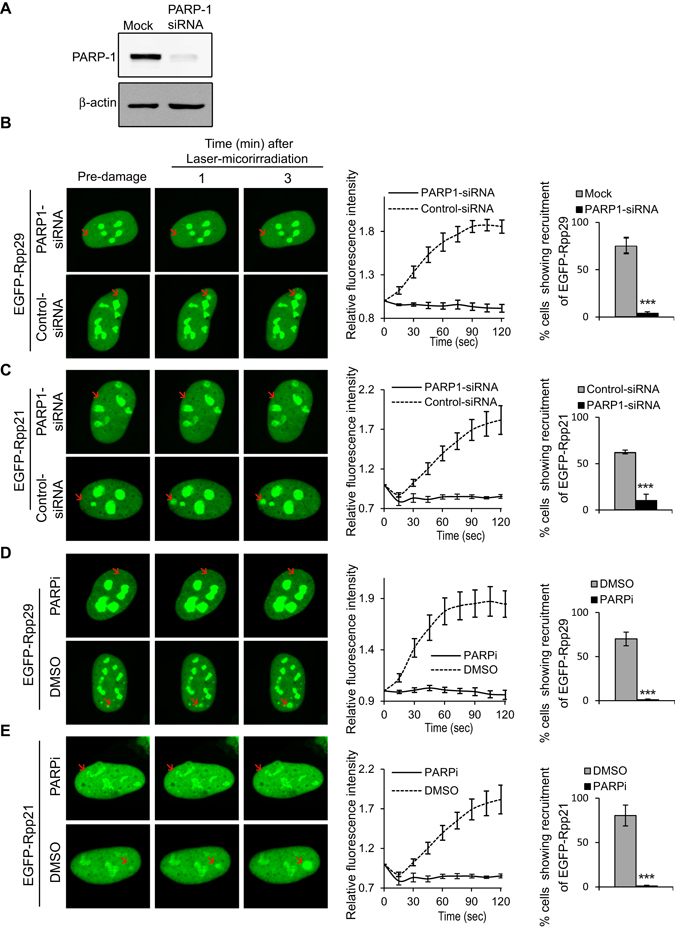
Rpp29 and Rpp21 are recruited to DNA damage sites in a PARP1-dependent manner. (**A**) Western blot analysis shows the efficiency of siRNA knockdown of PARP1. Protein extracts were prepared from mock and PARP1 siRNA-transfected U2OS cells and immunoblotted with PARP1 antibody. β-actin is used as a loading internal control. (**B**,**C**) Representative time-lapse images show subcellular distributions of EGFP-Rpp29 and EGFP-Rpp21 after laser microirradiation of mock and PARP1-depleted cells (Left). Line graphs (middle) depict fold increase in the relative fluorescence intensity of Rpp29 and Rpp21 at laser-microirradiated sites in mock and PARP1-siRNA transfected cells. Each measurement is representative of at least 10 cells. Error bars indicate SD. Column graphs (right) display the percentage of PARP1-depleted cells exhibiting accumulation of EGFP-Rpp29 and EGFP-Rpp21 at damage sites, as compared with mock-transfected cells. Error bars represent the SEM from two independent experiments. P-values were calculated by two-sided Student’s t-test relative to mock; ***p < 0.001. (**D,E**) Representative time-lapse images (left) show localization of EGFP-Rpp29 (**D**) and EGFP-Rpp21 (**E**) fusions to laser-microirradiated sites (marked by red arrow) in U2OS cells treated for 1 h with either DMSO or 5 μM of the PARP1/2 inhibitor (Ku- 0059436)(PARPi). Results shown are typical of two independent experiments and represent at least 30 different cells. Line graphs (middle) show fold increase in the relative fluorescence intensity of EGFP-Rpp29 and EGFP-Rpp21 at laser-microirradiated sites in untreated and PARPi-treated cells. Column graphs (right) display the percentage of PARPi-treated cells with accumulation of EGFP-Rpp29 and EGFP-Rpp21 at damage sites, as compared with untreated cells. Error bars express the SEM from two independent experiments. P-values were calculated by two-sided Student’s t-test relative to DMSO; ***p < 0.001.

As mentioned above, Rpp29 undergoes DNA damage-induced phosphorylation at serine 10 by PIKKs^53^. To find out if this site-specific phosphorylation is of any importance for the protein localization response to DSB, serine 10 residue was substituted with alanine, and recruitment of the resulted mutant Rpp29 protein to laser-microirradiated sites was examined. As shown in Supplementary Fig. S6, this PIKK-related mutation had no noticeable effect on Rpp29 compiling at damaged sites. Furthermore, inhibition of the ATM kinase activity by the pharmacological inhibitor KU-55933 did not influence the recruitment of Rpp29, as well as that of Rpp21, to laser-microirradiated sites (Fig. S7A,B). The efficacy of ATM inhibition was validated by visualizing CtIP at laser-microirradiated sites, in which this endonuclease marker was rather abolished (Fig. S7C), an outcome that is in agreement with a previous report^63^. Apparently, Rpp29 and Rpp21 recruitment to DNA damage sites is not regulated by ATM signaling pathway. Bioinformatics analysis revealed that Rpp29 and Rpp21 contain a consensus motif for binding of poly(ADP-ribose) (PAR) moieties (Fig. 4A). Indeed, bacterially expressed recombinant Rpp29 and Rpp21 human proteins, which were purified to near homogeneity, were able to bind PAR in a dose-dependent manner (Fig. 4B,C). Next, we sought to determine whether Rpp29 and Rpp21 undergo *in vivo* poly(ADP)-ribosylation (PARylation) in response to DNA damage. To do so, EGFP-Rpp29 and EGFP-Rpp21 fusions were purified using GFP-TRAP beads from undamaged and IR-damaged cells and the immunoprecipitates were immunoblotted with PAR and GFP antibodies. Results show that Rpp29 and Rpp21 were not PARylated (Fig. S8). Collectively, these observations suggest that binding of Rpp21 and Rpp29 to PAR moieties, rather than their PARylation, facilitates their mobilization to DNA damage sites. In agreement with this notion, two complementary approaches implicated PARP1 in the regulation of Rpp29 and Rpp21 recruitment to DNA breakage sites. First, depletion of U2OS cells from PARP1 by the use of siRNA (Fig. 5A) led to a remarkable decrease (~90%) in number of cells showing accumulation of Rpp21 and Rpp29 at laser-microirradiated sites, when compared with those of mock-transfected cells (Fig. 5B,C). Second, pretreating cells with PARP inhibitor Ku-0059436 abrogates the recruitment of Rpp29 and Rpp21 to DNA damage sites (Fig. 5D,E). Hence, PARP1 is critical for Rpp21 and Rpp29 recruitment to DNA damage sites.

### H1 RNA knockdown interferes with the recruitment of Rpp21 and Rpp29 to DNA damage sites

**Figure 6:**
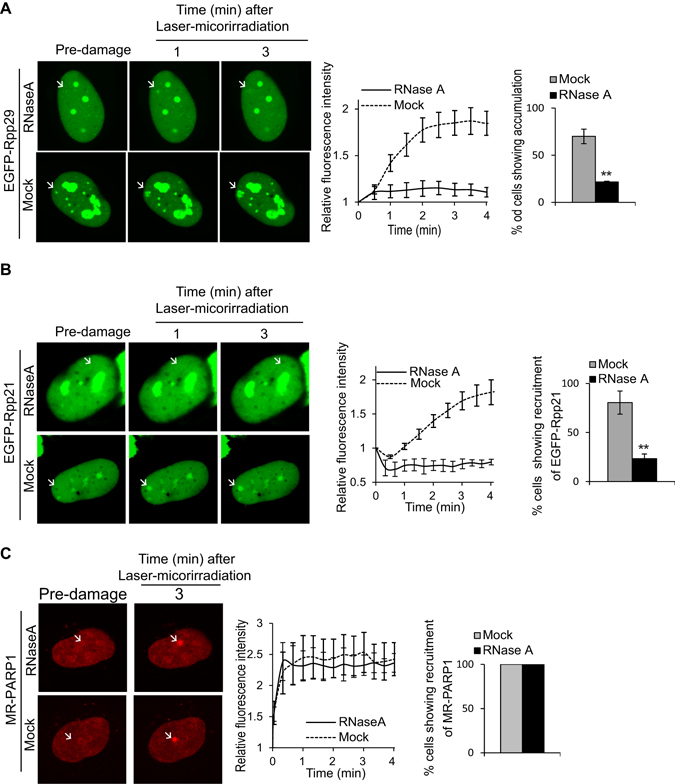
RNase A treatment of cells disrupts the recruitment of EGFP-Rpp29 and EGFP-Rpp21 to laser microirradiated sites. (**A**,**B**) Representative time-lapse images (left) of mock- and RNase A-treated U2OS cells showing the localization of EGFP-Rpp29 (**A**) and EGFP-Rpp21 (**B**) at the indicated times after laser-microirradiation of a single region marked by white arrow. Results shown are typical of three independent experiments and represent at least 30 different cells. Graphs (middle) show the increase in the relative fluorescence intensity of EGFP-Rpp29 and EGFP-Rpp21 at laser-microirradiated sites in mock and RNase A-treated cells. Each measurement is representative of at least 10 cells. Error bars indicate SD. Graphs (right) display the percentage of RNase A-treated cells showing subcellular distributions of EGFP-Rpp29 and EGFP-Rpp21, as compared with those seen in untreated cells. Error bars depicts the SEM from two independent experiments. P-values were calculated by two-sided Student’s t-test relative to DMSO; **p < 0.01. (**C**) As in A, except for that the laser microirradiation was applied on U2OS cells expressing MonomerRed-PARP1 (MR-PARP1).

As mentioned above, RNAs are implicated in recruiting DDR proteins to DNA damage sites. Since Rpp21 and Rpp29 can bind the catalytic H1 RNA of human RNase P^33, 38, 64^, we sought to assess whether RNA is required for these two proteins to be recruited to DNA damage sites. Thus U2OS-TetON cells expressing EGFP-Rpp21 or EGFP-Rpp29 were treated with RNase A^65^ and then were exposed to laser-microirradiation. RNase A treatment caused ~3.3-fold decrease in the number of cells (n = 30) that showed accumulation of Rpp29 and Rpp21 at DNA damaged sites (Fig. 6A,B). For comparison, RNase A treatment had no detectable effect on the recruitment of PARP1 to laser-microirradiated sites (Fig. 6C), thereby ruling out the possibility of a general indirect effect on the subcellular distribution of DDR proteins. Interestingly, inhibition of RNA Pol II activity by α-amanitin showed no detectable effect on the recruitment of EGFP-Rpp21 or EGFP-Rpp29 to DNA laser-microirradiated sites. Altogether, we concluded that RNA molecules, rather than active transcription, are critical for EGFP-Rpp21 or EGFP-Rpp29 accumulation at DNA damage sites (Fig. S9).

**Figure 7:**
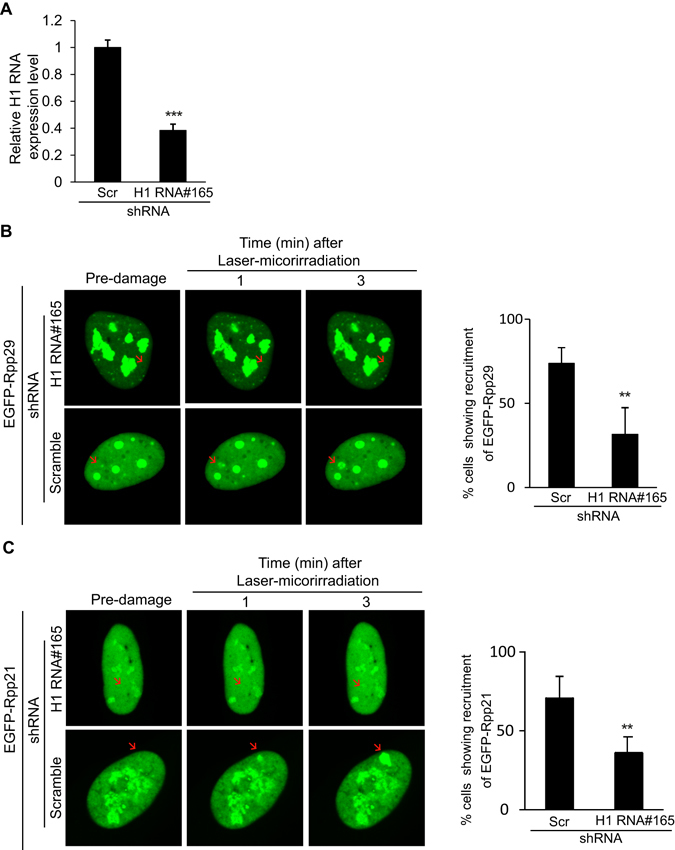
Knockdown of H1 RNA inhibits the recruitment of EGFP-Rpp29 and EGFP-Rpp21 to laser-microirradiated sites. (**A**) TaqMan-based Real-Time PCR shows ~65% knockdown of H1 RNA. RNA was extracted from U2OS cells transfected with either a scramble shRNA or H1 RNA shRNA#165. Real-time PCR was performed to measure H1 RNA level. The y-axis represents the relative RNA level of H1 RNA, which was normalized to that of GAPDH. Error bars represent the SEM from two independent experiments. (**B**,**C**) Recruitment of EGFP-Rpp29 (**B**) and EGFP-Rpp21 (**C**) to DNA damage sites cells with shRNA knockdown of H1 RNA or control cells expressing a scramble shRNA. Graphs display the percentage of cells exhibiting accumulation of the two fusion proteins at DNA damage sites. Error bars show the SEM from two independent experiments and represent at least 30 different cells. P-values were calculated by two-sided Student’s t-test relative to scramble shRNA; ** and *** indicates significance of p < 0.01 and 0.001.

To gain further insights into the identity of the RNA molecules that may regulate Rpp21 and Rpp29 recruitment to DNA damage sites, we checked the involvement of H1 RNA mainly because it associates with Rpp21 and Rpp29^32, 37–39^. Toward this end, H1 RNA was knocked down in U2OS-TetON cells by shRNA using lentivirus plasmid (pLKO.1-TRC). This shRNA was directed against a single-stranded RNA region spanning positions 164–184 of H1 RNA. Knock down of this relatively stable 340-nt RNA was determined by real-time qRT-PCR analysis. Remarkably, a reduction of ~65% in the steady state levels of H1 RNA (Fig. 7A) was accompanied by ~50% decrease in the number of cells with Rpp21 and Rpp29 localized at laser-microirradiated sites (Fig. 7B,C). Considering the knockdown efficiency of this stable RNA, these results indicate that H1 RNA is critical for accumulation of Rpp29 and Rpp21 at DSB sites, possibly through RNA-protein interactions.

### RNase P activity increases after DNA damage in a PARP1-dependent manner

**Figure 8:**
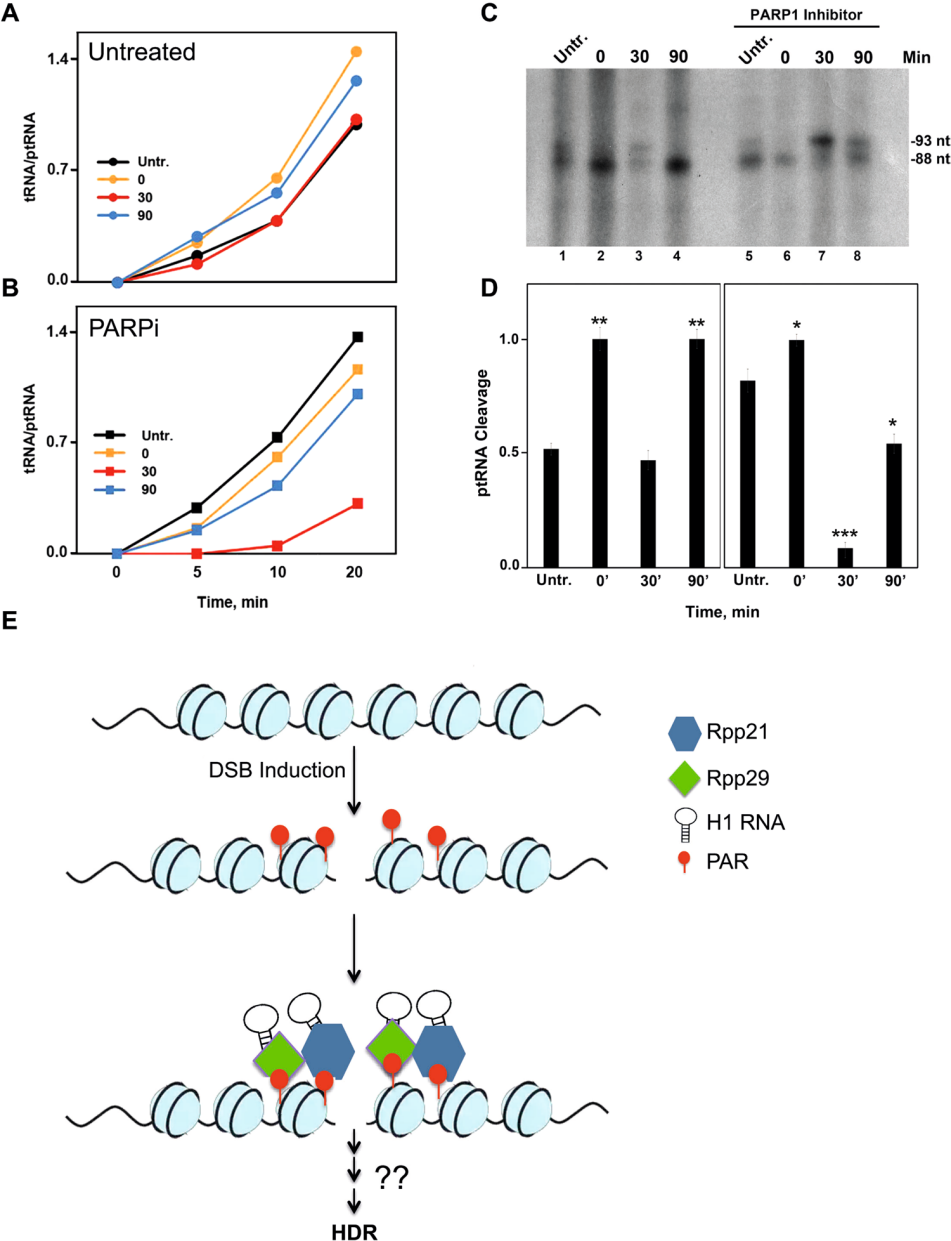
RNase P activity is induced after DNA damage in a PARP1-dependent manner. Whole cell extracts were prepared from untreated (**A**) and PARPi treated cells (**B**), and mature tRNA and ptRNA bands, seen in Supplementary Fig. S10, were quantified and ratios of product/substrate were plotted. (**C**) Processing of a nascent precursor tRNA^Arg^ (UCU) is inhibited by PARP1 inhibitor. U2OS cells were treated as in Supplementary Fig. S10, and dialyzed S20 extracts were assayed for processing of nascent precursor tRNA^Arg^ transcribed from a cloned gene for the indicated times (in min), as previously described^50^. Labeled RNAs were resolved in an 8% polyacrylamide sequencing gel. The positions of the primary transcript, 93 nt in length, and transcript processed at 5′ end by RNase P, 88 nt in size, are shown. Extracts of U2OS cells are not as efficient as HeLa cells in tRNA gene transcription and splicing to mature tRNA (not shown), thus producing weak labeled RNA signals. (**D**) The 93- and 88-nt transcript bands seen in C were quantitated and the ratios of processed to unprocessed tRNA^Arg^ were plotted. P-values were calculated by two-sided Student’s t-test relative to scr shRNA; *, **, and *** indicates significance of p < 0.05, 0.01 and 0.001, respectively. **(E)** A hypothetical model shows that local ADP-ribosylation at DSB site and H1 RNA molecule underpin the recruitment of Rpp29 and Rpp21 to promote HDR of DSBs.

The fact that H1 RNA is critical for Rpp29 and Rpp21 accumulation at DNA damage sites (Fig. 7) prompted us to check for RNase P activity in response to DNA damage. We found that the activity of this catalytic RNP was elevated in whole extracts (S20) prepared from U2OS cells that were harvested immediately after IR, as compared to that seen in extracts of untreated cells (Fig. 8A, 0 min; orange vs black graph; Fig. S10, A, lanes 3–6 versus 7–10). However, basal enzymatic activity was recovered in 30 min post irradiation (Fig. 8A). This swift and transient change in RNase P activity is consistent with the rapid recruitment of Rpp29 and Rpp21 to DNA damage sites and its dependence on H1 RNA. Moreover, PARP1 inhibition resulted in a marked decrease in RNase P activity in maturation of tRNA in whole extracts (Fig. 8B, orange vs black graphs; Fig. S10B, 11–14 vs S9A, lanes 11–14; 3′ tRNA band) and in coupled transcription/processing system of human tRNA^Arg^ (UCU) gene (Fig. 8C, lane 7 vs 3, and 8D) in extracts derived from irradiated U2OS cells, but not from untreated cells. The decrease was transient, as it was seen in 30 min post irradiation. Since the steady state levels of H1 RNA, as well as those of Rpp20 and Rpp40, remained unchanged in irradiated cells (Fig. S11), the observed PARP-dependent inhibition of RNase P activity and protein subunit recruitment to DSBs provide mechanistic basis for the involvement of a novel variant of nuclear RNase P RNP in DSB repair.

## Discussion

RNase P is classically defined as an RNA enzyme that carries out the excision of the 5′ leader of precursor tRNA. The catalytic asset of human RNase P is related to the H1 RNA subunit. Rpp29 and Rpp21 serve as cofactors for H1 RNA with which these two subunits form an active RNP complex^34, 38, 66, 67^. In this study, we have shown that Rpp29, Rpp21 and H1 RNA are involved in DDR. Functional analyses confirm that depletion of Rpp29 leads to accumulation of broken DNA, as determined by comet assay. Since other subunits, such as Rpp14, Rpp25 and Rpp38, do not similarly respond to the induction of DSBs, and the recruitment of Rpp21 and Rpp29 to DSB sites is independent of each other, but both subunits rely on H1 RNA, we speculate that a non-canonical form of RNase P responds to these lethal DNA lesions. In this regard, our data raise two possibilities: H1 RNA catalytic activity might be required for Rpp21 and Rpp29 recruitment to DSB sites. Alternatively, H1 RNA may serve as a scaffold for recruiting Rpp21 and Rpp29 subunits to DNA damage sites. In support of this, we know today about several ncRNAs such as: TSIX^68^, Air^69^, Tug1^70^, NRON^71^, Kcnq1ot1^72^ and HOTAIR^73^ which are able to interact with RNA binding proteins to regulate their activity and subcellular localization.

The dependence of recruitment and processing activity of RNase P on PARP1 indicates that this RNP participates in the major PARP1-mediated repair pathway. One challenging question is how does PARP1 regulate Rpp21 and Rpp29 recruitment to DNA breakage sites? Our data show that Rpp21 and Rpp29 possess PAR-binding motifs, bind PAR moieties *in vitro* (Fig. 4), but do not undergo ADP-ribosylation (Fig. S8). These observations altogether favor a model by which PARP1-mediated ADP-ribosylation of histones and non-histone proteins at sites of damage provide a platform for recruiting Rpp21 and Rpp29. Additional question: How is PARP1 involved in the DNA damage-induced increase of RNase P activity? While we cannot rule out a possibility that PARP1 may regulate RNase P activity by PARylating protein subunits other than Rpp21 and Rpp29, we assume that Rpp21/Rpp29 binding to PAR moieties may increase RNase P catalytic activity. In agreement with this assumption, several reports show that proteins activity is altered following their binding to PAR moieties^74^.

Our discovery that human RNase P has a role in DDR is supported by three indirect reports. First, Rpp29 undergoes IR-induced phosphorylation^53^. Second, Rpp29 interacts with histone H3.3 and represses its incorporation into chromatin^75^. H3.3 deposition is implicated in DDR, as previous studies reported on active deposition of H3.3 variant at UV-C damage sites^76^ and at laser microirradiation-induced DSB repair by NHEJ^77^. Third, genetic studies in Drosophila melanogaster with a mutated RNase P due to null mutations in the RPP30 gene, which codes for the core protein subunit Rpp30, reveal that sterile female display replication stress in atrophied ovaries^78^. The atrophied ovaries contain high steady state levels of precursor tRNAs with unprocessed 5′ leader sequences, but normal levels of mature tRNA. Though the molecular mechanism underlying replication stress remains unknown, it is proposed that structural and functional defects in RNase P lead to the activation of a number of key DNA damage checkpoint proteins, including p53, Claspin, and Chk2^78^.

Our results reveal that the role of human RNase P RNP in DSB repair is confined to HDR and not NHEJ. In general, Rpp29 and Rpp21 may promote HDR of DSBs through several pathways: (i) by serving as a scaffold for recruiting HDR proteins to DSB sites. (ii) By recruiting non-coding RNA molecules to DSB sites. In support of this, Rpp29 and Rpp21 are known to directly bind RNA^32, 37–39^. In addition, a growing number of evidence showed that RNA transcripts serve as a template for HDR of DSBs^79–81^. (iii) By processing noncoding RNA molecules in response to DNA damage. In this regard, tRNA-derived fragments have been shown to act as tumor suppressors through a posttranscriptional mechanism in human cells^82^. The tRNA-derived fragments are processed, by yet unknown enzyme(s), upon exposure to hypoxic stress^82^. Notably, previous studies reveal some other damage-induced RNAs processed to smaller RNAs by DICER and DROSHA or by DICER-like proteins^5, 10, 83^. On the other hand, CU1276, a tRNA derived 22-nt RNA that modulates DDR^84^ is generated in a DROSHA- and DICER-independent manner, suggesting that other RNA-processing machineries are implicated in tRNA processing^85–88^. Based on these studies, and based on our findings showing increased RNase P activity upon DNA damage, we hypothesize that human RNase P might be involved in DSB repair by generating stress-induced tRNA fragments. Future studies need to be conducted to elucidate whether indeed RNase P enzymatic activity is required for HDR of DSB.

In summary, our data identified a variant of human RNase P as a new component of DNA repairome that underpins HDR of DSBs (Fig. 8E). The remarkable evolutionary conservation of this RNP may shed new light on common primordial mechanisms by which eukaryotes preserve their genomes.

## Materials and Methods

### Cell culture, transfection and drug treatments

All cell lines used in this study were cultured in DMEM medium supplemented with 10% FBS, glutamine, streptomycin and penicillin. U2OS-TetON cells were cultured in the presence 200 µg/ml G418. Stable U2OS-TLR and U2OS-TetON-EGFP-Rpp29/Rpp21 cell lines were grown in media containing 0.6 µg/ml puromycin and 200 µg/ml G418. Cell transfection with plasmids was done by PolyJet transfection reagent (BioConsult) and with siRNA (Supplementary Table 3) by Lipofictamine2000 reagent (Invitrogen), according to the manufacturer’s instructions. Where indicated, cells were treated with 4 mM of Caffeine (Sigma; C0750), 1 µM of PARP inhibitor (Ku-0059436) for 1 h, 5 µM of ATM inhibitor (KU-55933) for 2 h, and 30 µg/ml α-amanitin (sigma) for 4 h. For RNase A experiments, cells were permeabilized with 0.5% or 2% Tween 20 in PBS for 10 minutes and then were treated with 1 mg/ml of RNase A dissolved in PBS for 15 min at room temperature^65^.

### Generation of U2OS-TetON-EGFP-Rpp29/Rpp21 cell lines

U2OS-TetON cell lines expressing EGFP-Rpp21 and EGFP-Rpp29 were established as previously described^58^. Briefly, EGFP-Rpp21 and EGFP-Rpp29 genes were sub-cloned into pTRE2/Puro vector (Clontech) and resulted constructs were introduced into U2OS-TetON cells (Clontech). Puromycin-resistant clones were then selected and tested for doxycycline (Dox)-induced expression of the fusion proteins by fluorescent microscopy. Cell clones that showed expression of the fusion proteins in the presence of Dox (Sigma, D9891) were selected for further characterization.

### Generation of U2OS-TLR cells

Viral particles containing the pCVL Traffic Light Reporter 1.1 (Sce target) Ef1a Puro^56^ were generated by transfecting HEK293T cells together with plasmids encoding the lentiviral proteins Gag, Pol and VSV-G. Media containing the viral particles were collected in 48 h post transfection, filtered and applied on U2OS cells. Infected cells with integrated reporters were selected in the presence of 0.6 μg/ml of puromycin for one week. Puromycin-resistant cells were sorted using FACSAria Cell Sorter (BD Biosciences) and mCherry negative cells were collected and used for TLR assays.

### Western blot

Hot-lysis buffer was used to prepare protein extracts. Samples were separated on SDS-PAGE gels and membranes were immunoblotted with relevant antibodies (Supplementary Table 1). The immunoblots were developed using Quantum ECL detection kit (K-12042-D20, Advansta). The intensity of the immunoblot bands was performed using ImageJ software.

### GFP Trap pull-down

GFP only, GFP-Rpp21, and GFP-Rpp29 fusions were purified from U2OS cells using GFP-TRAP methodology as previously described^58^. Briefly, U2OS cells were transfected with pEGFP-C1, pEGFP-Rpp21 or pEGFP-Rpp29 expression vectors and whole-cell extracts were prepared using NP40 lysis buffer and subjected to pull-down using GFP-Trap beads (Chromotek). Next, the immuno-complexes were washed, resolved by SDS-PAGE and blotted with the indicated antibodies.

### Immunofluorescence

Immunofluorescence analysis was done as previously described^58^. Cells were seeded on coverslips at least 24 h prior to the experiment, immunostained with the indicated antibodies (Table S1) and visualized using the inverted Zeiss LSM 700 confocal microscope with 40× oil EC Plan Neofluar objective. For 53BP1 foci quantification experiment, U2OS cells were seeded in 96-well plates 24 h prior experiment. Cells were exposed to 3 Gys of IR, fixed at the indicated time points and immunostained with 53BP1 antibody. High-content screening microscope (IN Cell Analyzer 2000; GE Healthcare) was used for automatic acquisition of at least 300 cells at each time point. The number of 53BP1 foci was calculated using the IN Cell Analyzer Workstation.

### RNA isolation, reverse transcription, and quantitative real-time PCR

RNA was extracted from U2OS cells transfected with siRNA/shRNA using TRizol reagent, according to the manufacturer’s instructions (Ambion). Aliquots of RNA (1 µg) were used for cDNA synthesis using the qScript cDNA Synthesis Kit (Quanta) using random primers. The steady state levels of mRNAs encoding for Rpp29 and Rpp21, as well as H1 RNA, were measured by real-time PCR in a Step-One-Plus Real time PCR System (Applied Biosystems) using Fast SYBR Green Master mix (Applied Biosystems) and specific primers for Rpp29, Rpp21, H1 RNA and GAPDH (Table S2). Each PCR reaction was repeated 3 times, data analysis and quantification were performed using the Step-One software V2.2 supplied by Applied Biosystems. RNA levels were calculated using ΔΔCt method from results that were normalized to GAPDH gene expression.

### Laser microirradiation

Cells were subjected to laser microirradiation as previously described^58^. Briefly, cells were plated on a 35-mm imaging dish with glass bottom (Ibidi; Cat#81158) and pre-sensitized with 10 µM of Hoechst 3334 dye for 15 min at 37 °C. Laser microirradiation was executed using a LSM-700 inverted confocal microscope equipped with CO_2_ module and 37 °C heating chamber. DNA damage was induced by microirradiating of a single region in the nucleus with 10 iterations of 405-nm laser beam. Time-lapse images were then acquired and intensity of fluorescence signals at laser-microirradiated sites were measured using a Zen 2009 software (Carl Zeiss).

### PAR-binding assay

Recombinant Rpp29 and Rpp21 human proteins were overexpressed in and purified from *Escherichia coli* BL21 strains, as previously described^38^. The soluble affinity purified His-tagged Rpp21 and Rpp29 proteins were tested for their ability to bind PAR moieties using the PAR-binding assay, as formerly specified^59^. Briefly, 1–5 pmol of recombinant Rpp29 and Rpp21 proteins were blotted onto a nitrocellulose membrane and blocked with TBST buffer supplemented with 5% milk. Radioactively labeled PAR moieties were produced by auto-modified PARP1 prepared by *in vitro* PARylation reaction. This reaction was carried out at room temperature for 20 min in a reaction buffer (50 mM Tris-HCl, pH 8, 25 mM MgCl_2_, 50 mM NaCl) supplemented with radiolabelled NAD^+^ (Perkin Elmer), activated DNA, and PARP1 enzyme (Trevigen). PAR moieties were detached from PARP1 using proteinase K and blotted membrane was incubated for 2 h with the radiolabelled PAR diluted in TBST buffer. Membranes were then washed with TBST, subjected to autoradiography and Western blotting using α-His and αH3 antibodies.

### TLR assay

TLR assay was performed as previously described^56, 89^. Briefly, U2OS-TLR cells were subjected to two sequential transfections with a time interval of 10 h. Cells were first transfected with siRNAs and then with two plasmids: pRRL-sEF1a HA.NLS.Sce(opt).T2A.IFP, expressing I-SceI fused to infrared fluorescent protein (IFP), and pRRL SFFV d20GFP.T2A.mTagBFP donor plasmid, expressing GFP donor sequence fused to blue fluorescent protein (BFP)^56^. Cells were harvested after 72 h and signals of GFP^+^ (reflecting HDR) and mCherry^+^ (reflecting NHEJ) were measured by four-color fluorescent flow-cytometry using a BD LSRFortessa™ cell analyser (BD Biosciences). A minimum of 10,000 double-positive (IFP^+^ and BFP^+^) cells were scored for each condition from three independent experiments. Results of treated cells were normalized to control cells. HDR values for each condition were normalized for percentage of cells at S and G2 phase monitored by propidium iodide-based standard flow-cytometry.

### Neutral comet assays

Comet assays were carried out with the Single Cell Gel Electrophoresis Assay-kit (Trevigen). U2OS cells were transfected with control or Rpp29 siRNAs. After 72 h of transfection, cells were exposed to 10 Gys of ionizing radiation from an X-ray machine (Faxitron, CellRad), followed by recovery for 1, 3 and 6 h. Approximately, 3 × 10^3^ cells were combined with molten LM Agarose at a ratio of 1:10 and then were applied onto comet slide for 30 min at 4 °C in the dark to solidify. Cells were lysed by incubation with lysis solution for 1 h at 4 °C and slides were run for 30 min at 13 volts. Slides were incubated for 30 min at RT with DNA precipitation solution and fixed with 70% ethanol for 30 min at RT. Samples were dried by incubation for 30 min at RT and stained with SYBR-green. Comet images were acquired using Nikon Eclipsed E400 Epi-fluorescence microscope and percentage of DNA in the comet tail was measured using commercially available COMET SCORETM (TriTeK Corporation) software.

### Transcription and processing of tRNA

Whole cell extracts (S20; 25 µl) were assayed for coupled transcription and processing of tRNA^Arg^, as previously described^48–50^. Transcription reactions (55-µl in volume) contained transcription buffer, rNTP and 10 µCi of [α-^32^P]UTP. In parallel, extracts were assayed for RNase P activity in processing of the 5′ leader of an internally ^32^P-labeled *S. pombe* precursor tRNA^Ser^ (p*SupS1*) or *E. coli* precursor tRNA^Tyr^ in 1 × MRP/TNET buffer containing 0.3 units of rRNasin, 150 ng of cold precursor tRNA and 10 mM DTT, as previously described^48–50^. Labeled RNAs were separated in denaturing 8% polyacrylamide/7 M urea gels that were then dried and exposed to autoradiography. Quantitation of the values of the RNA bands was done by the use of the EZQuant-Gel software for densitometric quantitation.

### Statistical analysis

Statistical analyses were performed using the demo version of GRAPHPAD prism software version.

## Electronic supplementary material

Supplementary Information
