# Supplementary material for: A role of human RNase P subunits, Rpp29 and Rpp21, in homology directed-repair of double-strand breaks
Source: Sci Rep. 2017 Apr 21;7:1002. doi: 10.1038/s41598-017-01185-6 (PMC5430778; doi:10.1038/s41598-017-01185-6)
Supplement: Supplementary file 1 — Supplementary Information [file 41598_2017_1185_MOESM1_ESM.pdf]

Supplementary Materials

A role of human RNase P subunits, Rpp29 and Rpp21, in homology directed-repair of double-strand breaks

Enas R Abu-Zhayia*[1], Hanan Khoury Haddad*[1], Noga Guttmann Raviv[1], Raphael Serruya[2], Nayef Jarrous[2] and Nabieh Ayoub[1]

[*] These authors contributed equally to this work

[1]Department of Biology, Technion - Israel Institute of Technology, Haifa 3200003, Israel.

[2]Department of Microbiology and Molecular Genetics, IMRIC, The Hebrew University-Hadassah Medical School, 91120 Jerusalem, Israel

Corresponding authors:

Nabieh Ayoub: Tel: +972-4-8294232; Fax: +972-4-8225153; Email: ayoubn@technion.ac.il

Nayef Jarrous: Tel: +972-2-6758233; fax: +972-2-6758808; e-mail: jarrous@md.huji.ac.il

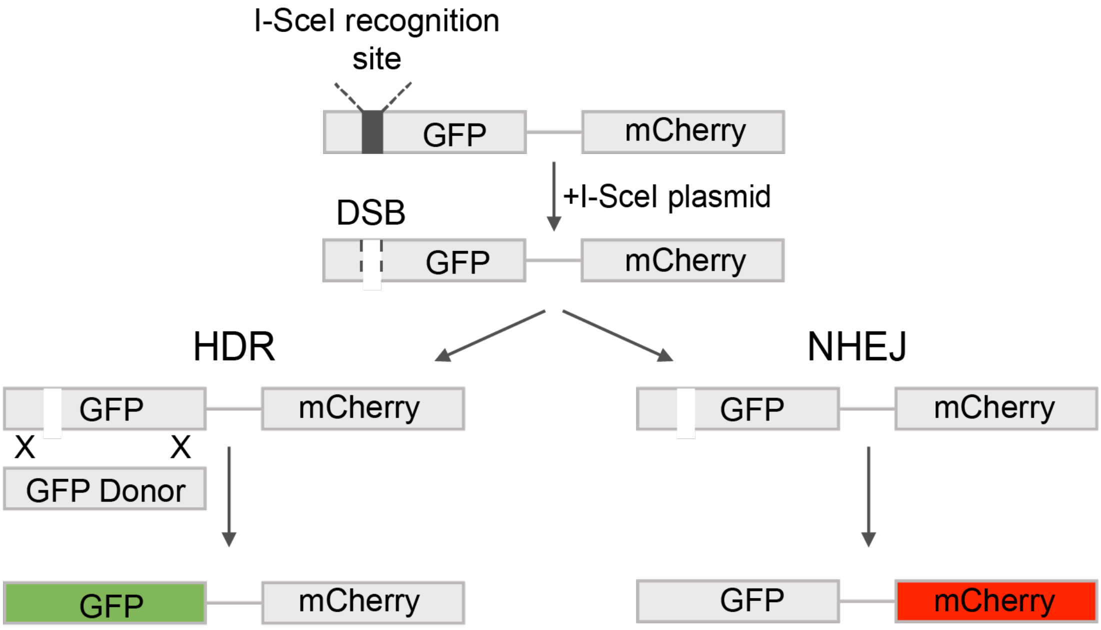

Figure S1: Schematic of the traffic light reporter (TLR) system. U2OS-TLR cell line contains an I-Sce-I recognition site integrated within a GFP-reporter cassette and out-of-frame mCherry gene. Based on this system, transient expression of the I-Sce-I protein fused to infra-red fluorescent protein (IFP) and an exogenous GFP donor gene fused to blue fluorescent protein (BFP) will enable the simultaneous evaluation of DSB repair by HDR and NHEJ in the same cell. Repair of the DSB induced by I-SceI endonuclease through HDR pathway will restore the functionality of the GFP gene and cells will be green. Error-prone repair of the DSBs by NHEJ will introduce a frame-shift within the out-of-frame mCherry gene, thus leading to the expression of mCherry, and cells will be red.

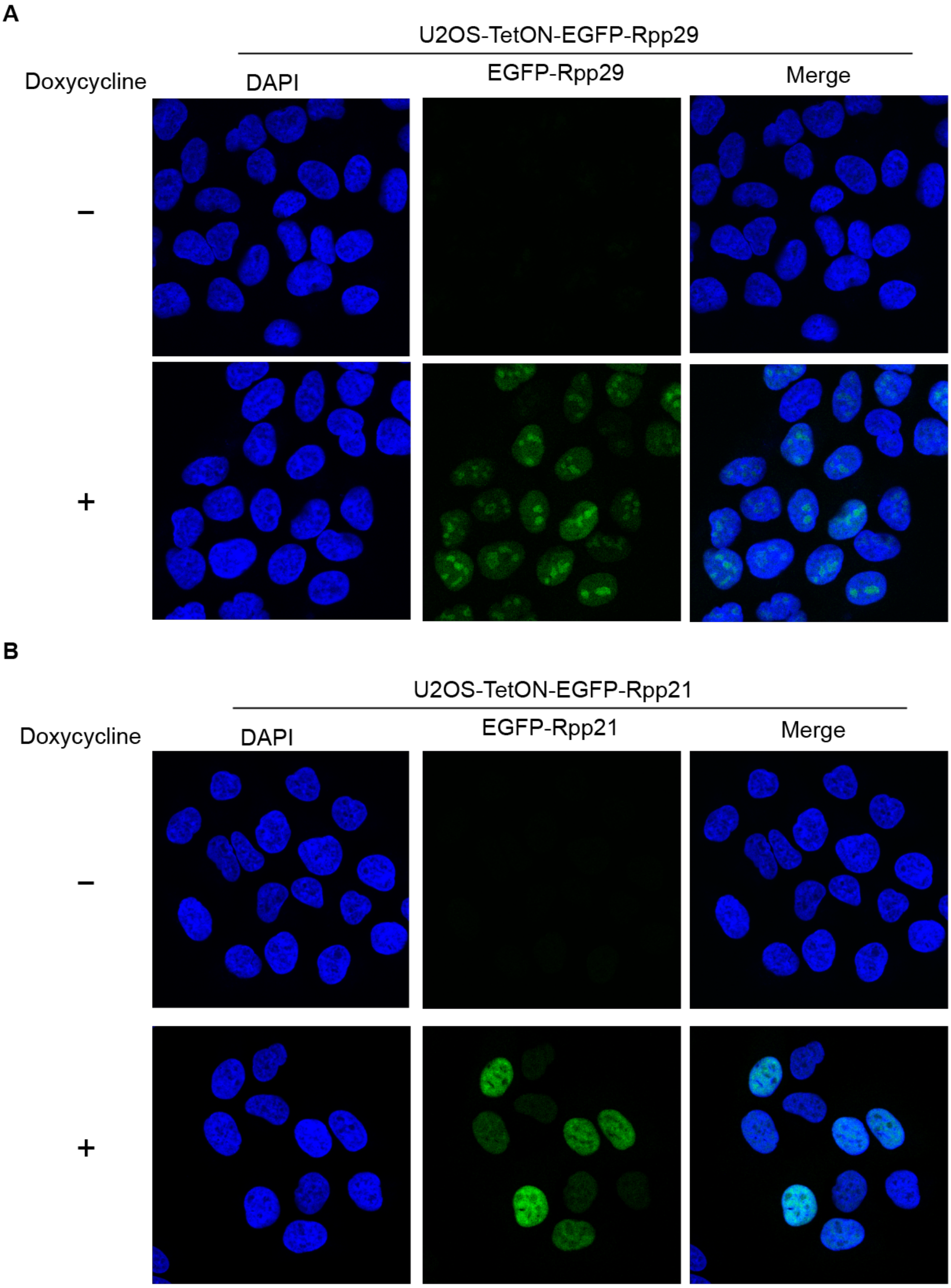

Figure S2. U2OS-TetON-EGFP-Rpp29 and U2OS-TetON-EGFP-Rpp21 cell lines that express EGFP-Rpp29 and EGFP-Rpp21 upon addition of doxycycline. (A-B) Representative fields of mock- and doxycycline-treated U2OS-TetON-EGFP-Rpp29 (A) and U2OS-TetON-EGFP-Rpp21(B) cells fixed and stained with DAPI (blue). EGFP-Rpp29 and EGFP-Rpp21 are in green colors.

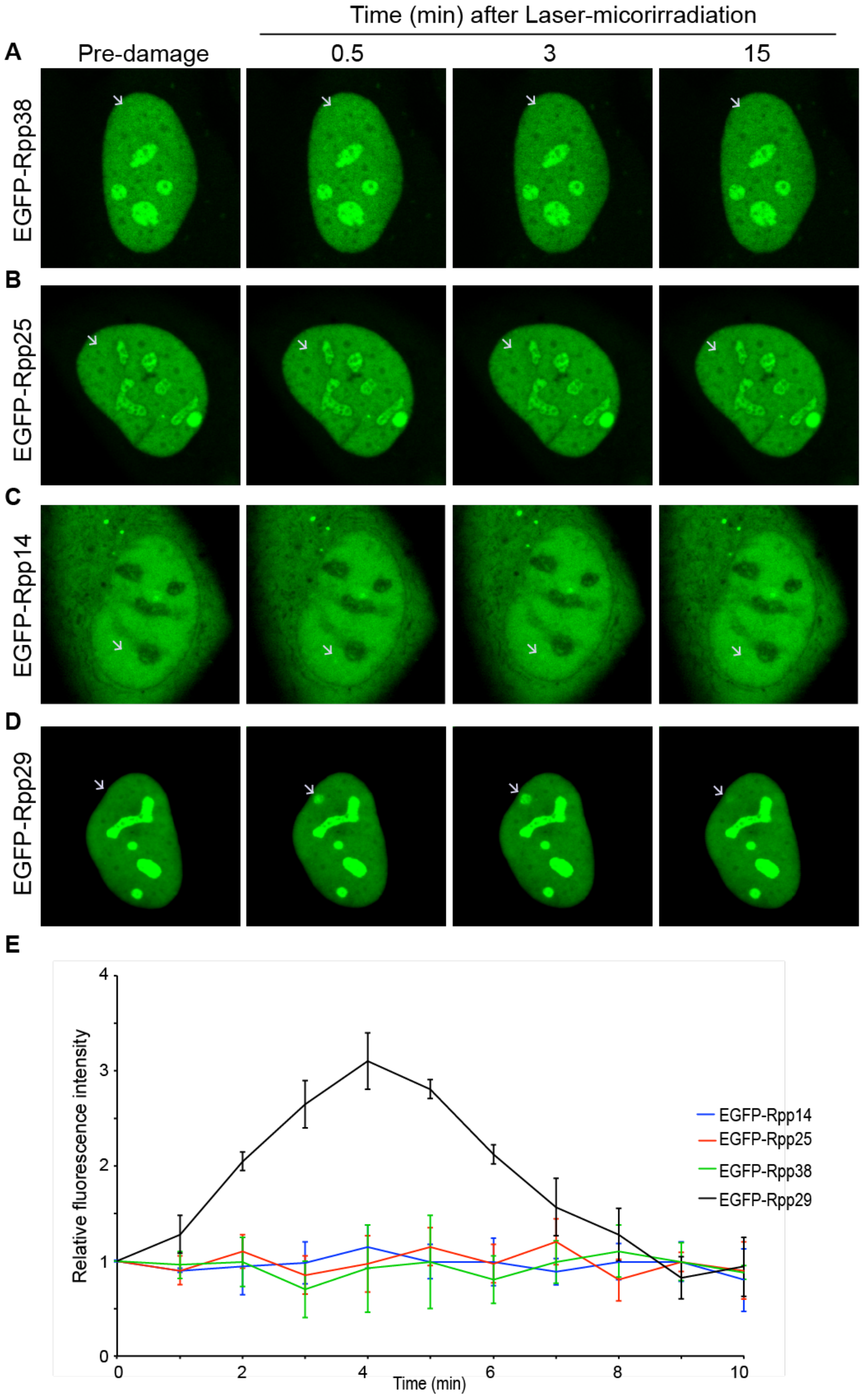

Figure S3. Rpp38, Rpp25 and Rpp14 are not recruited to laser microirradiated sites. (A-D) Time-lapse images show the subcellular distribution of EGFP-Rpp38 (A) EGFP-Rpp25 (B), EGFP-Rpp14 (C) and EGFP-Rpp29 (D) at the indicated time points after laser microirradiation of a single region indicated by a white arrow. **(E)** Graph shows the relative fluorescence intensity of the indicated fusion proteins at laser microirradiated sites. Each measurement is representative of at least 15 cells. Error bars indicate standard deviation (SD).

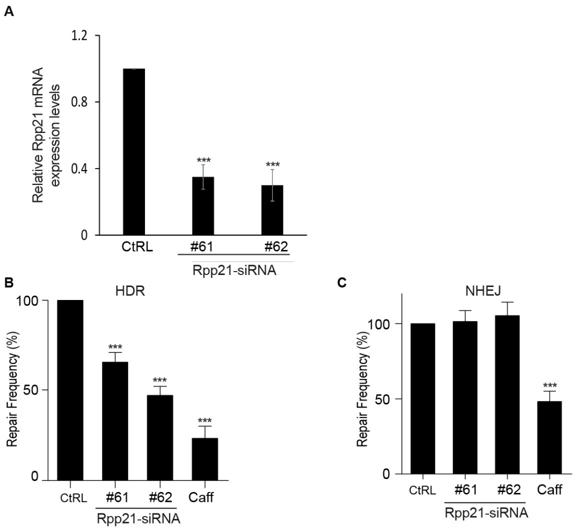

Figure S4. Rpp21 promotes homology-directed repair of double-strand breaks. **(A)** Quantitative real-time PCR shows the efficiency of Rpp21 knockdown in U2OS-TLR cells. Cells were transfected with control siRNA (Ctrl) or two different Rpp21 siRNAs; #61 and #62. The y-axis represents the relative Rpp21 mRNA level normalized to that of GAPDH. **(B-C)** Rpp21 knockdown impairs HDR, but not NHEJ, of DSBs generated by I-SceI endonuclease. U2OS-TLR cells were transfected with Rpp21 siRNA and subjected to TLR assay as described in Figure 2. Results shown are typical of three independent experiments. Error bars represent SD. P-values were calculated by two-sided Student's t-test relative to Ctrl siRNA; *** p<0.001

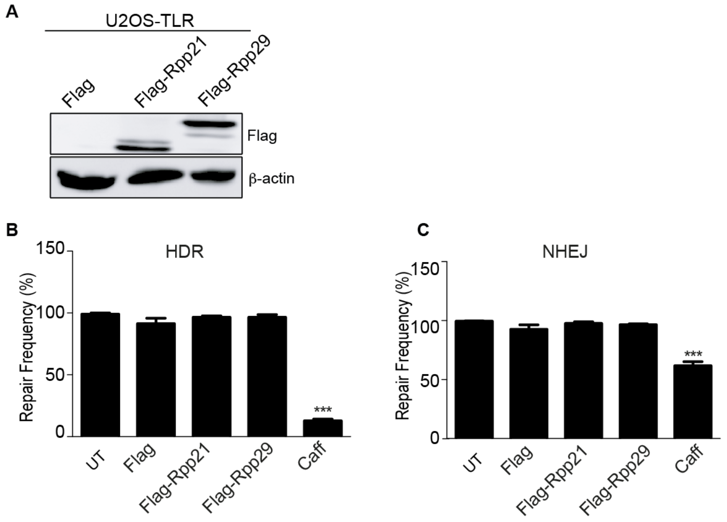

Figure S5. Overexpression of Rpp21 and Rpp29 has no detectable effect on the integrity of DSB repair.

(A) Western blotting shows overexpression of flag-Rpp21 and flag-Rpp29 fusions in U2OS-TLR cells. Protein extracts were prepared from U2OS-TLR cells transfected with expression vectors encoding flag-Rpp21, flag-Rpp29 or flag only and immunoblotted with the indicated antibodies. **(B-C)** TLR assay shows that overexpression of Rpp21 and Rpp29 has no detectable effect on the integrity of DSB repair. U2OS-TLR cells expressing flag-Rpp21, flag-Rpp29 or flag only were subjected to TLR assay as described in Figure 2. P-values were calculated by two-sided Student's t-test relative to Ctrl siRNA; *** p<0.001.

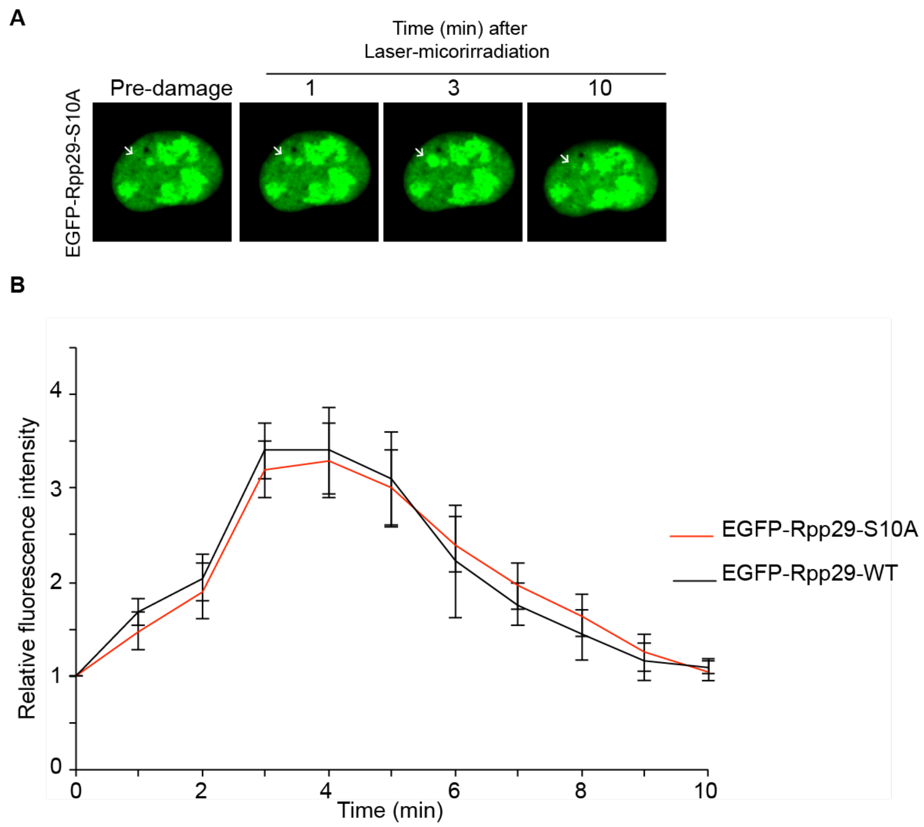

Figure S6. Rpp29-S10A mutant is recruited to laser-microirradiated sites
(A) Representative time-lapse images show the recruitment of EGFP-Rpp29-S10A mutant to laser microirradiated sites. **(B)** Graph depicts fold increase in the relative fluorescence intensity of EGFP-Rpp29-WT and EGFP-Rpp29-S10A at laser-microirradiated sites. Each measurement is representative of 10 cells. Error bars indicate SD.

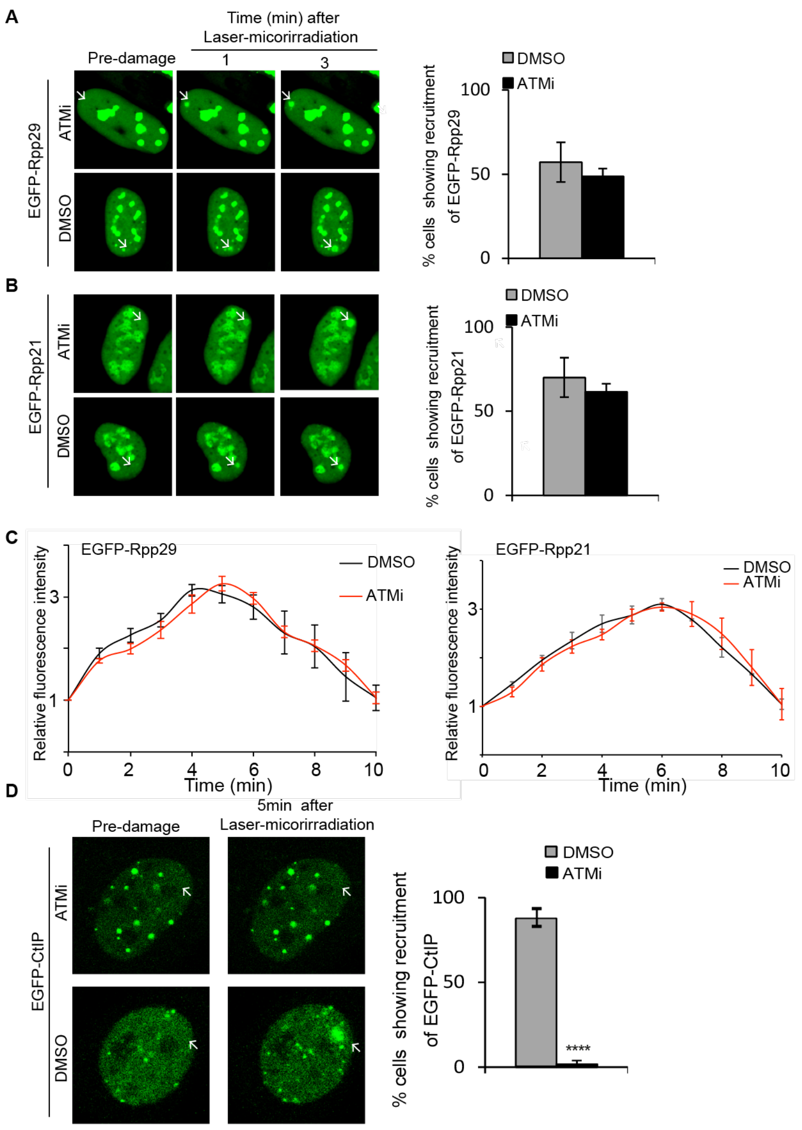

Figure S7. Rpp29 and Rpp21 recruitment to laser microirradiated sites are independent of ATM kinase activity. (A-B) Time-lapse images show the effect of ATM chemical inhibition on the recruitment of EGFP-Rpp29 (A) and EGFP-Rpp21 (B) to sites of DNA damage, marked by white arrow. U2OS cells were treated with 20µM of ATM inhibitor KU-55933 for 2h before laser microirradiation. Error bars indicate SD of 10 different cells. Column graphs (right) display the percentage of ATMi-treated cells exhibiting accumulation of EGFP-Rpp29 and EGFP-Rpp21 to DNA damage sites, as compared with DMSO-treated cells. (C) Graphs depicts fold increase in the relative fluorescence intensity of GFP-Rpp29 and GFP-Rpp21 at sites of DNA damage, in mock, and ATMi treated cells. (D) Time-lapse images show the effect of ATM chemical inhibition on the recruitment of EGFP-CtIP to laser microirradiated sites. U2OS cells were treated with DMSO or 20µM of ATM inhibitor KU-55933 for 2h before laser microirradiation. Column graphs (right) display the percentage of ATMi-treated cells exhibiting accumulation of EGFP-CtIP to DNA damage sites, as compared with DMSO-treated cells. Error bars represent the SEM from two independent experiments. P-values were calculated by two-sided Student's t-test relative to DMSO; **** $p<0.0001$

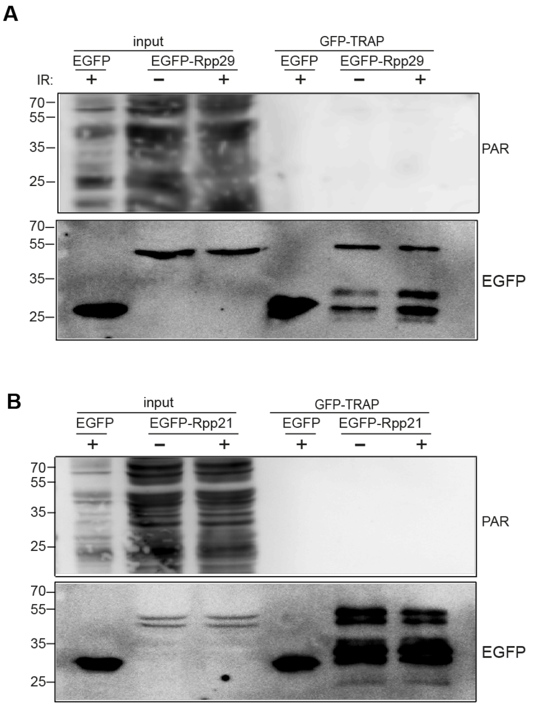

Figure S8. GFP-Rpp21 and GFP-Rpp29 are not PARylated. (A) U2OS cells were transfected with expression vectors encoding EGFP only, EGFP-Rpp21 or EGFP-Rpp29 were exposed to 10Gy of IR, subjected to GFP-TARP pull down, and immunoblotted using PAR antibody (*Upper*). Next, the membranes were stripped and immunostained with GFP antibody (*Lower*).

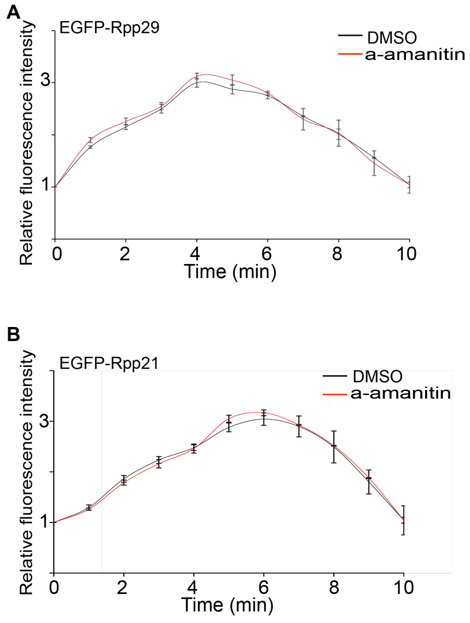

Figure S9. α-amanitin treatment has no noticeable effect on Rpp21 and Rpp29 recruitment to DNA damage sites. (A-B) U2OS cells were treated with DMSO or 30μg/ml of α-amanitin for 4h before laser microirradiation. The graphs show fold increase in fluorescence intensity of EGFP-Rpp29 (A) and EGFP-Rpp21 (B) at laser-microirradiated sites. Results shown are typical of 2 independent experiments (n=10 cells). Error bars indicate SD.

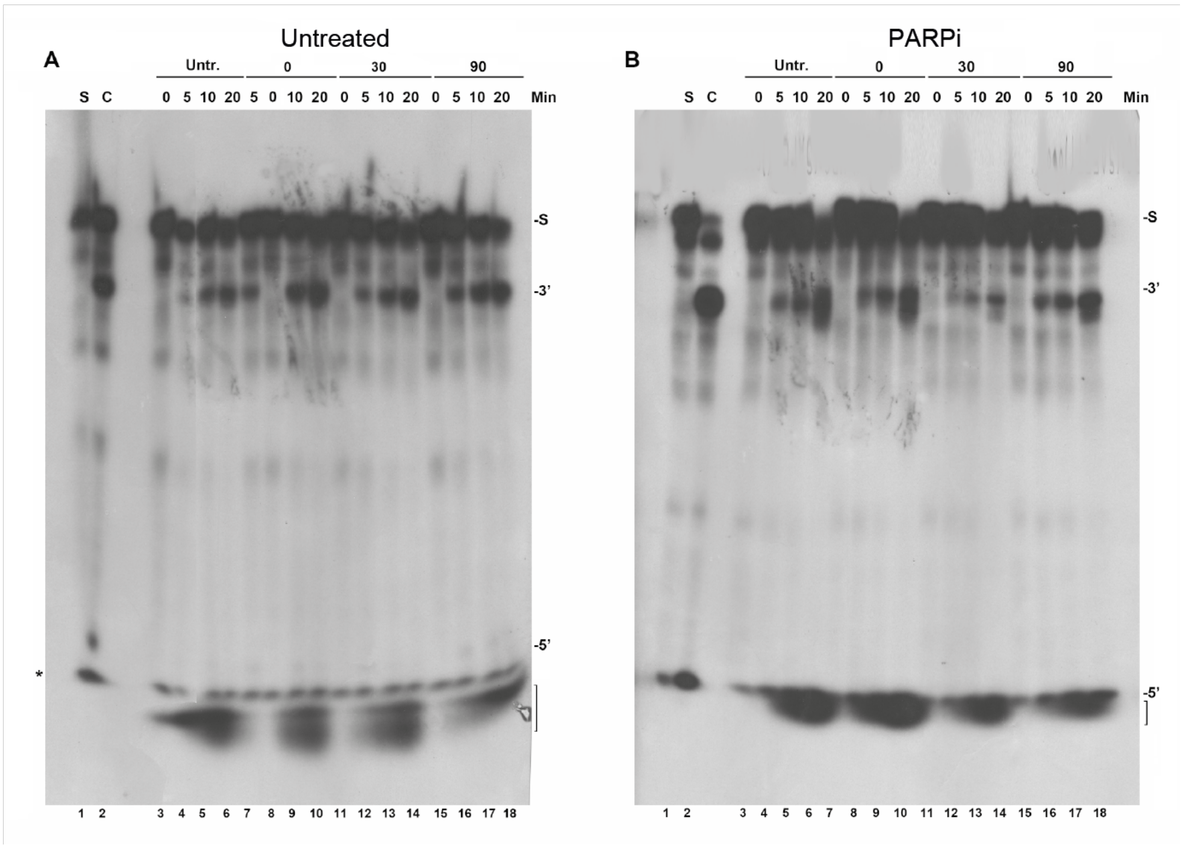

Figure S10. PARP1-dependent RNase P activity in response to DSB damage. U2OS were left untreated (**A**) or treated with PARP1 inhibitor (Ku-0059436)(**B**). After 24 h, cells were left without irradiation (Untr.) or were exposed to γ-irradiation for 10 sec, and were followed for recovery for 0, 30 and 90 min, as indicated. Whole cell extracts were prepared and assayed for processing of an internal ^{32}P-labled precursor tRNA^{Ser}. Cleavage products were analyzed in an 8% denaturing polyacrylamide gel. Precursor tRNA (S), mature tRNA (3'), 5' leader (5') and degraded small RNAs (bracket) were revealed by exposure of the dried gel to autoradiography. In **A**, an empty space between lane 4 and 5 was deleted for presentation of the kinetic analyses. Lanes 2 and 3 represent intact precursor tRNA (S) and standard cleavage control (C) by a DEAE-purified human HeLa RNase P.

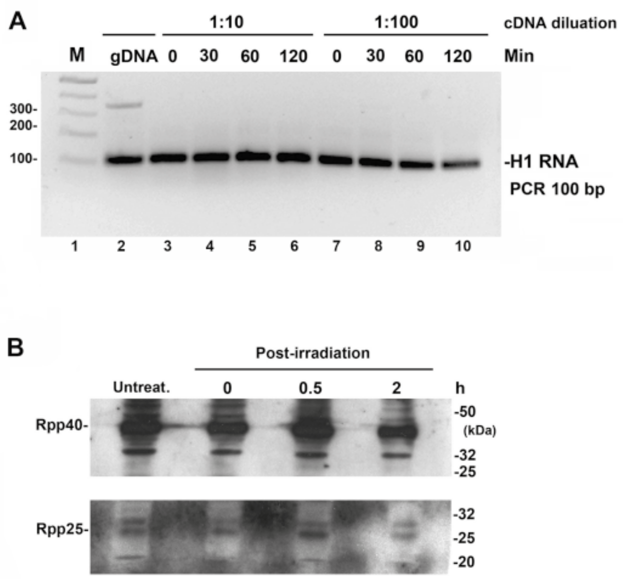

Figure S11. Steady state levels of H1 RNA and protein subunits of human RNase P remained unchanged in response to DSB. (**A**) RT-PCR analysis of H1 RNA in γ-irradiated U2OS cells. Total RNA was extracted from irradiated cells, recovered for the indicated times (lanes 3-6) and subjected to RT-PCR using forward and reverse primers amplifying the first 100 nt of H1 RNA. Two dilutions of 1:10 and 1:100 of the cDNA preparations were included to validate reaction specificity. The 100-bp PCR product is shown. gDNA, positive PCR using genomic DNA as template. M, DNA size marker. (**B**) Western blot analysis of Rpp25 and Rpp40 in extracts obtained from γ-irradiated U2OS cells harvested at the indicated times after recovery. A protein size marker (in kDa) is shown.

Supplementary Table 1. Antibodies used in this study.

Name	Source	Dilution for WB	Dilution for IF
**Primary antibodies**			
Anti-β-actin	Sigma A5441	1:10000	
Anti-PARP1	Enzo ALX-210–895-R100	1:3000	
Anti-H3	Abcam ab1791	1:2,000–20,000	
Anti-γH2AX (ser139)	Millipore 05-636		1:2500
Anti-γH2AX (ser139)	Cell signaling 2577	1:1000	
Anti-RPA32/RPA2 [9H8]	Abcan ab2175	1:500	
Anti-pRPA32 (S4/S8)	Bethyl A300-245A	1:4000	
Anti-pKAP1 (S824)	Bethyl A300-797A	1:5000	
Anti-53BP1	Bethyl A300-272A		1:1,000
Anti Rpp21/29	(Jarrous et al.,2001)		1:200
Anti GFP	Abcam ab290	1:1000	
Anti Poly-ADP-ribose binding reagent	Millipore MABE1031	1:4000	
Anti pNBS1	Abcam ab47272	1:1000	
Anti Flag	Sigma F1804	1:1000	
**Secondary antibodies**			
Anti-mouse(IgG)-HRP	Amersham	1:10,000	
Anti-rabbit(IgG)-HRP	Jackson ImmunoResearch 111–035-003	1:20,000	
Donkey anti-rabbit-Alexa Fluor 488	Invitrogen A21206		1:500
Donkey anti-mouse-Alexa Fluor 568	Invitrogen A10037		1:500

Supplementary Table 2. Primers used in this study.

Sequence	Primer
GCTCGGGAGCCAAGCAGATT	Rpp21-F
GGAACCCTGAGTCTGCATTT	Rpp21-R
AACCAGAGCAGCAGAGATAC	Rpp29-F
TGGCTGCGTGTCTGGCTTGA	Rpp29-R
TTGCCGGAGCTTGGAACAGA	H1 RNA-F
GCGGAGGAGAGTAGTCTGAA	H1 RNA-R
GCTCCAATTCCCCATCTCA	GAPDH-F
ACCCTTACACGCTTGGATGAA	GAPDH-R

Supplementary Table 3. siRNAs used in this study.

**siRNA**	**Sequence (sense)**
Rpp29 #23	GGGAGCUGCGGCUCUUUGACAUUAA
Rpp29 #24	GGCCAAGCUCUUAAAGGCAGAUAUU
Rpp29 #25	ACUGUGGAAACCGAUGGCUUUAUUU
Rpp21 #61	GGACCAUUGCGAAGCGGCUCGUCUU
Rpp21 #62	CAGACCUGCCUAACAUGCCAGCGCA
PARP1	CCAUCGAUGUCAACUAUGTT
